# Supersaturated Gel Formulation (SGF) of Atorvastatin at a Maximum Dose of 80 mg with Enhanced Solubility, Dissolution, and Physical Stability

**DOI:** 10.3390/gels10120837

**Published:** 2024-12-19

**Authors:** Jin Woo Park, Sa-Won Lee, Jun Hak Lee, Sung Mo Park, Sung Jun Cho, Han-Joo Maeng, Kwan Hyung Cho

**Affiliations:** 1 College of Pharmacy and Inje Institute of Pharmaceutical Sciences and Research, Inje University, 197 Inje-ro, Gimhae 50834, Republic of Korea; wlsdn4361@naver.com (J.W.P.); dlwnsgkr2341@gmail.com (J.H.L.); msk2241@gmail.com (S.M.P.); 2 Department of Pharmaceutical Engineering, Inje University, 197 Inje-ro, Gimhae 50834, Republic of Korea; lsw314@inje.ac.kr; 3 Department of Anesthesiology, National Medical Center, 245 Eulji-ro, Seoul 04564, Republic of Korea; csj8756@gmail.com; 4 College of Pharmacy, Gachon University, 191 Hambakmoei-ro, Yeonsu-gu, Incheon 21936, Republic of Korea; hjmaeng@gachon.ac.kr

**Keywords:** atorvastatin calcium, organogel formulation, dissolution, solubility, physical stability

## Abstract

The objective of this work was to develop a supersaturated gel formulation (SGF) loaded with the maximum atorvastatin calcium trihydrate (ATR) dose. The maximum dose strength of ATR needs to be reduced through improving solubility and dissolution rate to mitigate side effects due to the necessity of taking high doses. ATR has highly pH-dependent solubility at 37 °C, leading to poor solubility (<10 μg/mL) in stomach acid (pH 1.2). Among the various molecular weights of polyethylene glycols (PEGs) and surfactants, PEG 200 and d-α-tocopheryl polyethylene glycol 1000 succinate (TPGS) were selected as the solubilizer and precipitation inhibitor for ATR, respectively. PEG 200 demonstrated very high solubility for ATR (>60%, *w*/*w*), and the combined use of TPGS and PEG 200 formed an organogel state and suppressed ATR precipitation, showing 15-fold higher dispersion solubility in buffer solution at pH 1.2 compared to the formulation with PEG 200 alone. The optimal SGF composition (ATR/PEG 200/TPGS = 10/60/30, *w*/*w*) exhibited an over 95% dissolution rate within 2 h at pH 1.2, compared to less than 50% for the original commercial product. In a transmission electron microscope analysis, the SGF suppressed ATR precipitation and revealed smaller precipitated particles (<300 nm) compared to the control samples. In the XRD analysis, the SGF was physically stable for 100 days at room temperature without the recrystallization of ATR. In conclusion, the SGF suggested in this work would be an alternative formulation for the treatment of dyslipidemia with enhanced solubility, dissolution, and physical stability.

## 1. Introduction

**Figure 1:**
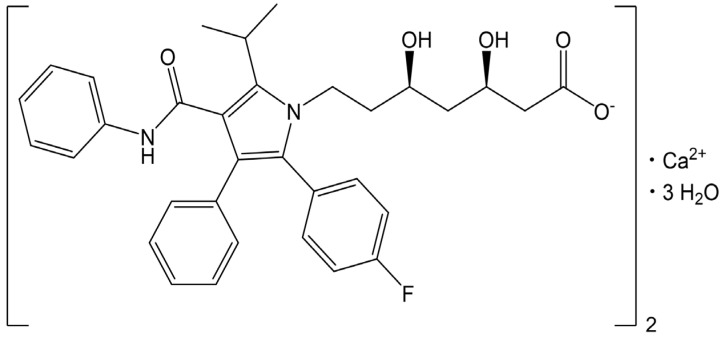
Chemical structure of atorvastatin calcium trihydrate.

Atorvastatin calcium trihydrate (ATR) is a commercially available drug form, as shown in Figure 1, and is a highly effective β-hydroxy β-methylglutaryl-CoA (HMG-CoA) reductase inhibitor that is widely prescribed for the treatment of both familial and non-familial dyslipidemia [1]. ATR functions as a selective and competitive HMG-CoA reductase inhibitor and has been recognized as the most popular statin for reducing low-density lipoprotein and total cholesterol levels in the blood [2,3]. Given the rising prevalence of dyslipidemia, a primary risk factor for cardiovascular diseases such as ischemic heart disease and stroke, effective cholesterol management is paramount [4].

Despite its significant therapeutic potential, ATR’s oral bioavailability is limited to approximately 12% due to its low aqueous solubility, instability in acidic pH, and pronounced first-pass hepatic metabolism [5]. The commercially available ATR product, Lipitor^®^ (Pfizer, New York, NY, USA), is formulated in oral tablet form across doses of 10, 20, 40, and 80 mg. There is an eight-fold difference between the minimum and maximum doses. Patients with severe dyslipidemia require the highest dose (up to 80 mg daily) to achieve therapeutic efficacy, though this simultaneously heightens the risk of side effects such as myopathy, rhabdomyolysis, and hepatotoxicity in a dose-dependent manner [5,6,7]. While high-dose ATR reduces mortality from cardiac events, it paradoxically raises the risk of non-cardiac mortality [6]. ATR’s poor solubility in aqueous media restricts its dissolution and absorption in the gastrointestinal tract, increasing the maximum dose up to 80 mg as atorvastatin free form [3,8,9]. Consequently, there is a fundamental need for advanced formulations that enhance ATR’s dissolution rate, solubility, and overall bioavailability, which leads to a reduction in maximum dose strength and the mitigation of side effects.

Various approaches, including nano-sizing, salt formation, co-crystallization, and self-emulsifying drug delivery systems (SEDDSs), have been explored to enhance ATR’s dissolution rate and bioavailability [10,11,12,13]. However, many of these strategies face limitations. For instance, amorphous drugs produced via the supercritical antisolvent process are prone to recrystallization in aqueous environments, which negatively impacts their solubility and bioavailability [10]. SEDDS formulations often suffer from low drug-loading capacities; previous formulations contained only 1.9% ATR by weight [11], while solidified SEDDS formulations with mannitol had a drug loading of less than 2.0% [12]. Co-crystal formulations can face stability challenges, especially in high-humidity conditions, impacting their long-term physical stability [13].

Supersaturated technology has gained attention as a viable strategy to improve water-insoluble drugs’ solubility, dissolution rate, and gastrointestinal absorption [14]. Achieving and maintaining supersaturation involves solubilizing a water-insoluble drug beyond its saturated intrinsic solubility while preventing its precipitation [15,16]. Commonly used precipitation inhibitors, such as hydroxypropyl methylcellulose (HPMC), hydroxypropylmethylcellulose-acetate succinate (HPMCAS), and polyethylene glycol (PEG), increase viscosity and adhere to crystal surfaces to kinetically and thermodynamically block recrystallization [14]. Notably, d-α-tocopheryl polyethylene glycol 1000 succinate (TPGS) has shown efficacy in stabilizing supersaturated states for drugs like indomethacin, paclitaxel, and celecoxib, preventing crystallization and precipitation [17,18,19].

The gel formulation exists in a semi-solid state characterized by the loss of flowability and is utilized for various applications, including oral, transdermal, and topical delivery; sustained-release injections; ophthalmic applications; diagnostics; and surgical assistance. Gel formulations are generally classified into hydrogels and organogels, which possess an aqueous continuous phase and an organic continuous phase, respectively. Although the application of organogels as pharmaceutical formulations has gained less attention in medication fields compared to hydrogels, they have potential advantages in terms of solubilization and physicochemical stabilization [20]. For example, organogel-based formulations facilitate oral, intravenous, and transdermal routes of administration for hydrophobic medications, such as statins [21,22,23]. The broad functionality range of organogels arises from the combination of properties given by the organic liquid and gelator components. The properties and functionalities of organogels are varied due to the wide range of organic liquids and compatible gelators available [24].

Preventing drug recrystallization in supersaturated formulations, particularly at room temperature, is critical. Gel formulations offer advantages in this regard, as increased viscosity restricts molecular mobility, thereby enhancing physical stability relative to fluid formulations [25]. Moisture protection can be achieved by incorporating ATR into a gel matrix, while encapsulating supersaturated semi-solid gels in hard gelatin capsules provides a stable and convenient dosage form [26]. Thus, supersaturated gel formulations (SGFs), which maintain high ATR concentrations in the gastrointestinal tract, represent a promising and physically stable drug delivery strategy that enhances dissolution and bioavailability [27].

In this study, we aimed to develop an SGF capable of effectively loading high ATR concentrations (>10%). To achieve a gel state, we used PEG with varying molecular weights as a solubilizer and TPGS as a precipitation inhibitor. The organogel SGFs were characterized for dispersion solubility in buffer solutions with pH 1.2 (to mimic the acidic stomach environment) and for dissolution rates across various pH levels, and for precipitation inhibition during dissolution using TEM analysis. Additionally, the capacity of these SGFs to prevent recrystallization during long-term storage was evaluated through an X-ray diffraction (XRD) analysis with the ultimate goal of optimizing therapeutic efficacy and offering an improved treatment option for dyslipidemia management.

## 2. Results and Discussion

### 2.1. pH Solubility of ATR at 37 °C

The solubility of ATR in buffer solutions with pH values of 0.1 N HCl/NaCl (pH 1.2), 0.05 M acetate (pH 4.0), and 0.05 M potassium phosphate-buffered solution (pH 6.8), and in water, was determined at a body temperature (37 °C), and the results are detailed in Table 1. The solubility of ATR was the highest at pH 6.8, measuring 158.35 ± 4.58 μg/mL, and it decreased to 12.81 ± 1.16 μg/mL at pH 4.0 and further to 6.63 ± 0.24 μg/mL at pH 1.2. In water, its solubility was 66.30 ± 0.89 μg/mL. The increased solubility at pH 6.8 was due to the dissociation of the terminal carboxyl groups in ATR’s molecular structure, which has a lower pKa of 4.5, as shown in Figure 1. This dissociation led to the formation of negative charges at that pH [28]. Conversely, ATR was practically insoluble at pH 1.2 and 4.0 because the major species of ATR was in a non-ionized form, leading to very low solubility, below 10 μg/mL. ATR showed highly pH-dependent solubility (pH 6.8 > water >> pH 4.0 and pH 1.2), and a high dose of ATR required improvement in solubility at pH 1.2 and pH 4.0.

### 2.2. Solubility of ATR in PEGs

**Figure 2:**
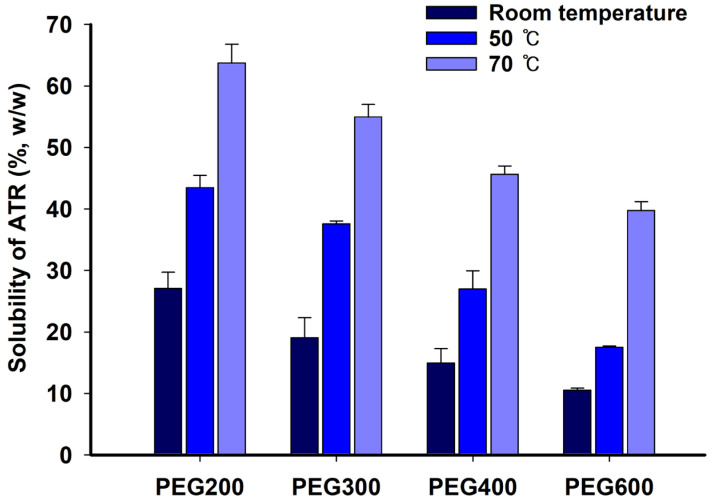
Solubility of ATR in PEG at room temperature, 50 °C, and 70 °C.

To select an appropriate PEG molecular weight as a solubilizer, the solubility of ATR in PEGs was measured and summarized in Figure 2. The solubility of ATR significantly increased with rising temperature, and its solubility at 70 °C was more than twice as high as that at room temperature. Additionally, ATR showed higher solubility at each temperature with the low-molecular-weight PEG. ATR had a high affinity with PEG and exhibited high solubility, with the low-molecular-weight PEG 200 showing a solubility of over 60% (*w*/*w*) at 70 °C. The solubility of ATR was measured at 70 °C, resulting in 63.73 ± 3.07%, 54.94 ± 2.06%, 45.63 ± 1.35%, and 39.71 ± 1.45% for PEG 200, PEG 300, PEG 400, and PEG 600, respectively. These values are considerably high, showing that low-molecular-weight PEG 200 was a very effective solubilizer. The supersaturated ATR in PEG at a higher temperature, namely 70 °C, maintained the solution state without precipitation or recrystallization after cooling at room temperature. There were two reasons for ATR’s decrease in solubility with PEG’s increasing molecular weight. First, the affinity with ATR molecules decreased as the molecular weight increased. Second, the increase in molecular weight was accompanied by an increase in viscosity, which reduced the rate at which ATR molecules dissolved, diffused, or were released from the powder surface. Therefore, low-molecular-weight PEG 200 was selected as an effective solubilizing agent for ATR over high-molecular-weight PEGs.

### 2.3. Dispersion Solubility of ATR in PEGs

**Figure 3:**
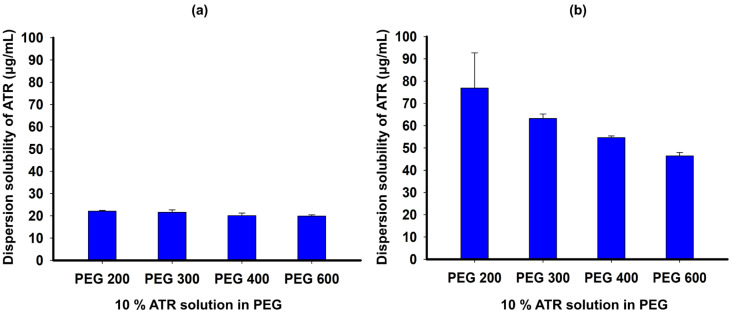
Dispersion solubility of ATR in PEG solution with pH 1.2 buffer (**a**) and pH 4.0 buffer (**b**).

To evaluate the ability of PEG to maintain ATR supersaturation, we tested the dispersion solubility of ATR in PEGs in pH 1.2 and pH 4.0 buffer solutions, as shown in Figure 3. The dispersion solubility of a 10% ATR solution in PEG 200, PEG 300, PEG 400, and PEG 600 in pH 1.2 buffer solution was 22.04 ± 0.41, 21.57 ± 1.14, 20.07 ± 1.17, and 19.83 ± 0.61 μg/mL, respectively (Figure 3a). In pH 4.0 buffer solution, the ATR solution in PEG 200 had the highest dispersion solubility of 76.83 ± 15.86 μg/mL, which is 10-fold higher than the solubility of ATR at pH 1.2, while the solutions in PEG 300, PEG 400, and PEG 600 had lower dispersion solubility with increasing molecular weights of PEGs with values of 63.20 ± 2.04, 54.61 ± 0.75, and 46.42 ± 1.51 μg/mL, respectively (Figure 3b). The ATR molecules in the PEG solution exhibited increased precipitation and reduced supersaturation levels when dispersed in an acidic medium, correlating with the increased molecular weight of PEG. These findings align with the solubility data observed for PEGs with varying molecular weights. The ability of PEG to induce ATR supersaturation appeared insufficient, particularly under low pH conditions. This was likely due to PEG’s inadequate adsorption onto the drug crystal surface, which failed to effectively inhibit crystal growth in ATR [29].

### 2.4. Solubility of ATR in Surfactants

**Figure 4:**
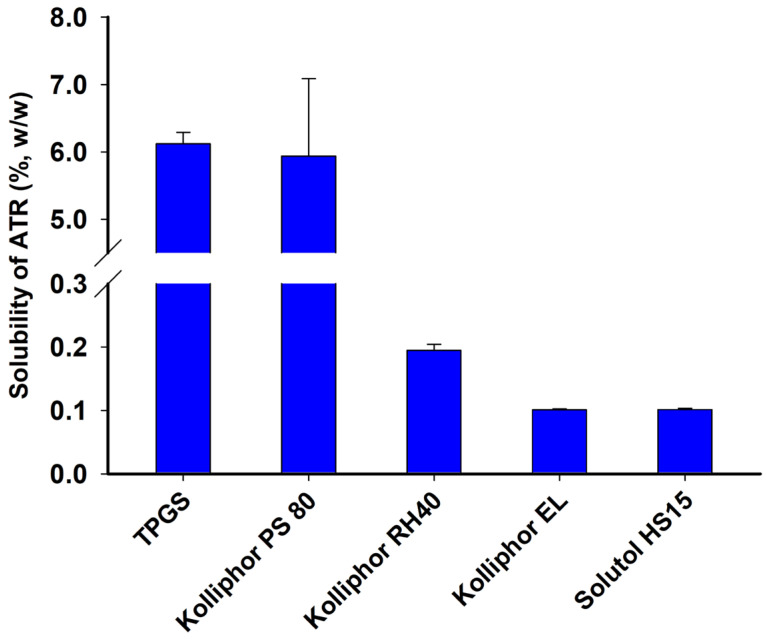
Solubility of ATR in surfactant at 70 °C.

The solubility of ATR at 70 °C was determined, and the results are summarized in Figure 4. ATR demonstrated an average solubility exceeding 5% in TPGS and Kolliphor PS80, whereas its solubility was below 0.3% in other surfactants such as Kolliphor RH40, Kolliphor EL, and Solutol HS15. The solubility of ATR was found to be 6.12 ± 0.17%, 5.94 ± 1.15%, 0.19 ± 0.01%, 0.11 ± 0.01%, and 0.10 ± 0.01% in TPGS, Kolliphor PS80, Kolliphor RH40, Kolliphor EL, and Solutol HS15, respectively. TPGS was chosen as a precipitation inhibitor in an acidic medium due to its high molecular affinity with ATR and its high solubility. Surfactants like TPGS can effectively function by surrounding ATR molecules when they encounter an acidic medium and disperse, thereby inhibiting recrystallization or precipitation.

### 2.5. Solubilized Ratio of ATR and the Dispersion Solubility of ATR Formulations

**Figure 5:**
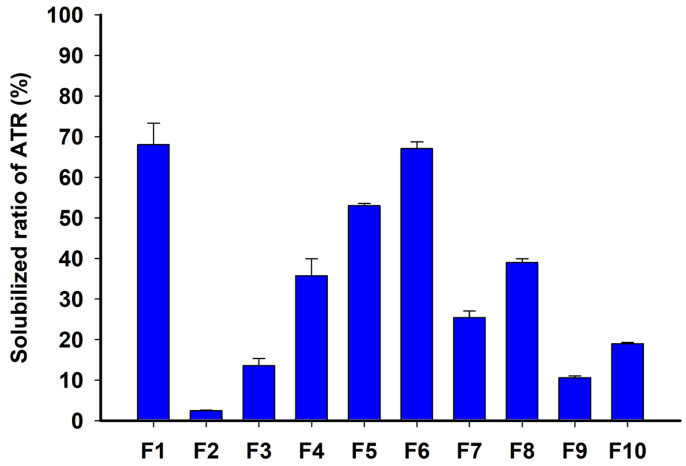
Solubilized ratio of ATR in dispersion solubility of ATR formulations in pH 1.2 buffer.

Dispersion solubility tests were conducted using ATR formulations, and the solubilized ratio of ATR over the total ATR amount in formulations was measured and summarized in Figure 5. F1, with a high content of 95% TPGS and 5% ATR loading, exhibited a high solubilized ratio of 67.98 ± 5.33%, indicating that TPGS prevented ATR precipitation and improved solubilization in the acidic aqueous phase. Similarly, as the composition% of TPGS increased, the solubilized ratio of ATR between F2 and F6 with 10% ATR loading were increased to 2.44 ± 0.15%, 13.56 ± 1.77%, 35.66 ± 4.22%, 52.97 ± 0.57%, and 67.00 ± 1.69%, respectively. Among these, F6 demonstrated an almost equivalent solubilized ratio to F1 while containing twice as much ATR loading at 10%. This suggested that PEG 200 and TPGS interacted to solubilize ATR at high concentrations within the formulation and suppressed ATR precipitation when dispersed in an acidic medium, thereby synergistically increasing the solubilized ratio. This could be explained by PEG 200 microenvironmentally creating a co-solvent when it encountered the aqueous phase, while TPGS inhibited crystal growth through micelle formation [30]. F7–F10, with 20% or 30% ATR loading, showed an average solubilized ratio of less than 40% due to high ATR loading. High ATR loading % in the formulation promoted precipitation during dispersion in the aqueous medium, significantly reducing ATR solubilization. A high loading % of ATR relative to the formulation vehicle was a factor that promoted precipitation in the microenvironment [29]. In summary, F6 was selected as the formulation with the highest ATR solubilization and supersaturation capability.

### 2.6. Physical Properties (Visual Observation, Differential Scanning Calorimetry (DSC), and Gel Strength)

**Figure 6:**
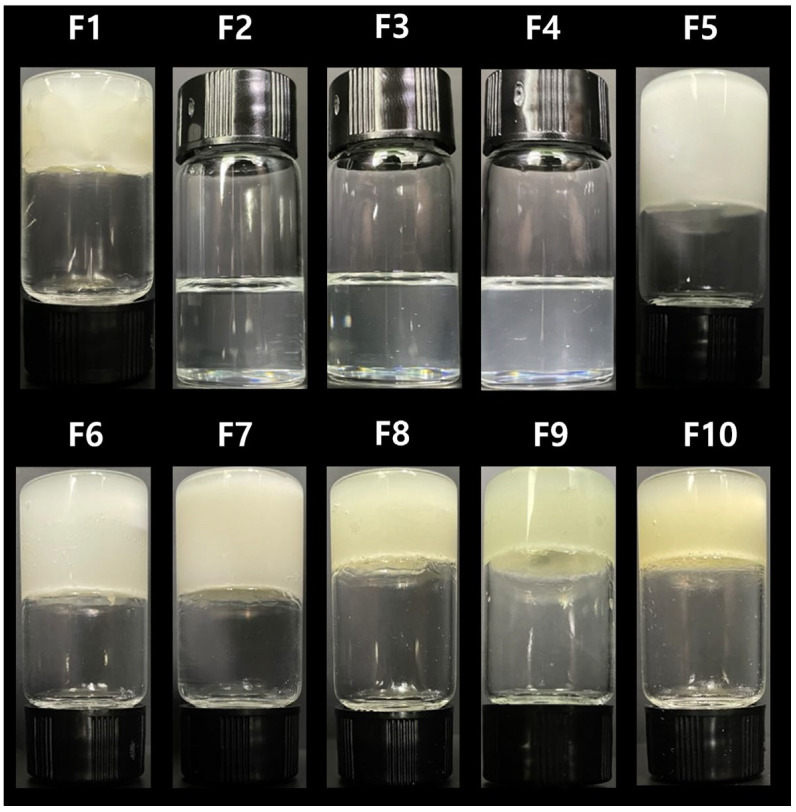
The observed visual images of the ATR formulations.

Images of the prepared ATR formulations (F1–F10) in a vial are shown in Figure 6. F1 was solid due to its high loading % of TPGS (95%). In F2–F6, with the same loading % of ATR and an increased weight ratio of TPGS to PEG 200, F2–F4 exhibited a liquid state, while F5 and F6 were in a gel state. When ATR and TPGS were at ≥10% and ≥20%, respectively, the formulation became a gel and lost flowability. F7–F10, with ATR loading of ≥20%, all exhibited a gel state. Although PEG 200 was a liquid, its combination with the total solid amount of ATR and TPGS at ≥30% resulted in a gel state. Among the gel formulations, a higher ratio of ATR or TPGS led to a firmer gel, with ATR having a greater gelling effect than TPGS.

**Figure 7:**
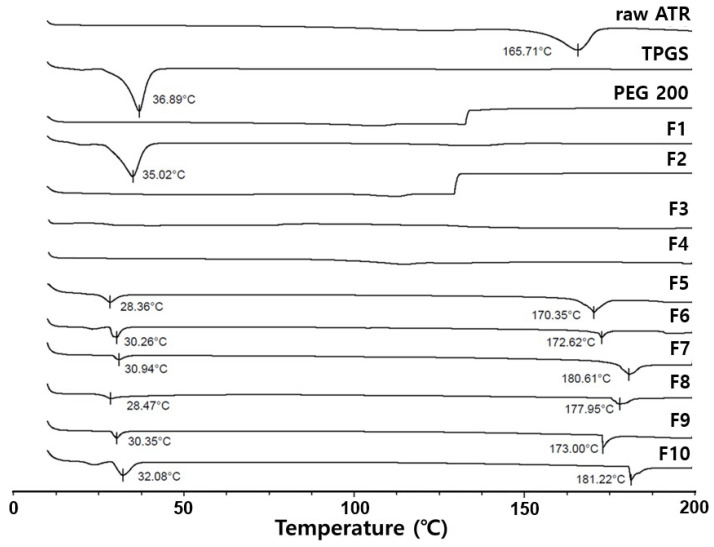
DSC curve of raw ATR, TPGS, PEG 200, and Formulations (F1–F10).

Figure 7 displays DSC curves for raw ATR, TPGS, PEG200, and formulations (F1 to F10). Raw ATR and TPGS showed intrinsic peak melting points at 165.65 °C and 36.89 °C, respectively. Due to its high TPGS content, F1 exhibited a peak melting point similar to pure TPGS. However, F5–F10, containing both PEG and TPGS showed a peak melting point between 28 °C and 32 °C, lower than that of TPGS. This indicates that the unique melting point of TPGS shifted to a lower temperature due to the influence of ATR and PEG 200, and the size and area of the endothermic peak decreased. Furthermore, F5–F10 exhibited a second peak between 170 °C and 182 °C, which was derived from ATR, but did not indicate the presence of crystalline ATR in the gel state. This is because the second melting point was significantly higher than ATR’s intrinsic melting point (161.71 °C), suggesting that amorphous ATR likely recrystallized during heating and its recrystallized form melted [31].

**Figure 8:**
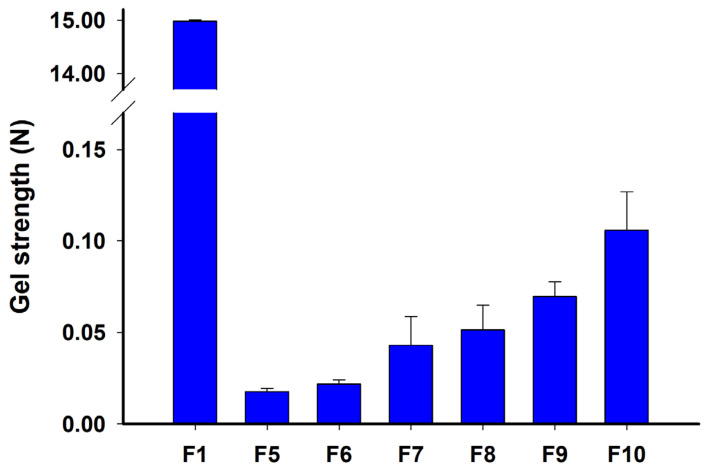
Gel strength of ATR formulations: F1, F5, F6, F7, F8, F9, and F10.

As shown in Figure 8, F1, composed of solid ATR and TPGS, showed a high gel strength of 14.980 ± 0.029 N, indicating a mostly solid state. In contrast, F5–F10 exhibited low values of gel strength with 0.017 ± 0.002 N (F5), 0.022 ± 0.002 N (F6), 0.043 ± 0.016 N (F7), 0.051 ± 0.014 N (F8), 0.069 ± 0.008 N (F9), and 0.106 ± 0.021 N (F10), indicating a semi-solid or gel state. It was also confirmed that the influence of the ATR content on gel strength was greater than that of TPGS. Therefore, the prepared F2–F4 were classified as supersaturated liquid formulations (SLF), and F5–F10 were classified as supersaturated gel formulations (SGFs) called organogels. These gel or liquid formulations were expected to have better dispersibility in aqueous phases compared to solids, and the gels would particularly reduce the mobility of dissolved molecules, potentially inhibiting recrystallization through molecular rearrangement compared to liquid formulations [32].

### 2.7. Dissolution Test

**Figure 9:**
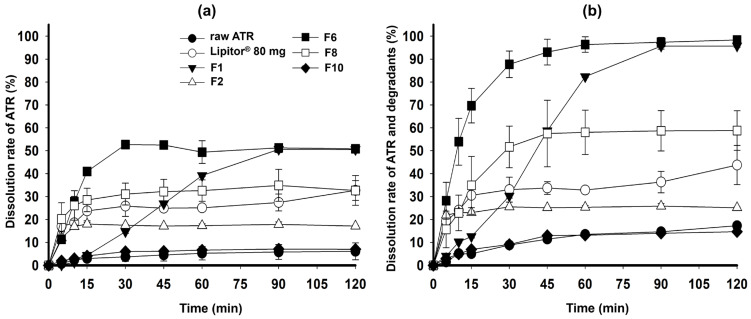
Dissolution profiles of raw ATR, Lipitor^®^ 80 mg, F1, F2, F6, F8, and F10 in pH 1.2; dissolution rate of ATR (**a**) and total ATR and degradants (**b**).

In Figure 9a, the dissolution rate of raw ATR was very poor at less than 10% until 120 min passed. Among the formulations, F6 exhibited the highest dissolution rate of 52.68 ± 1.02% at 30 min, which was approximately twice as high as the commercial 80 mg Lipitor^®^ tablet (25.83 ± 4.47%). Although F1, containing only TPGS, showed a similar dissolution rate to F6 at 120 min, it demonstrated a slower dissolution rate at 90 min. TPGS was inherently in a solid state at room temperature due to its crystal form, which slowed down the dispersion and disintegration of the formulation [33]. F2, containing only PEG 200, showed a dissolution rate of less than 20% due to ATR precipitation. Depending on the loading % of ATR, the final dissolution rate was in the order of F6 > F8 >> F10. This result is consistent with the solubilized ratio of ATR, as shown in Figure 5; as the ATR loading % increased, the dissolution rate decreased due to ATR precipitation. Overall, the formulations commonly exhibited a final dissolution rate of less than 60% due to the instability of ATR in acidic solutions, leading to the formation of major degradation materials (imp-A1, Imp-H, and Imp-J). Therefore, these major degradation materials were identified and quantified in the HPLC analysis together with ATR, and the dissolution rates are shown in Figure 9b. The dissolution rates of all formulations increased, but the dissolution profiles remained similar even when including the degradation materials. Notably, for F6 in the pH 1.2 medium—which was expected to have the lowest ATR solubility and dissolution rate—the dissolution rate was 96.33 ± 3.37% at 60 min, which is the highest dissolution rate among all formulations, showing significantly improved results compared to the commercial product.

**Figure 10:**
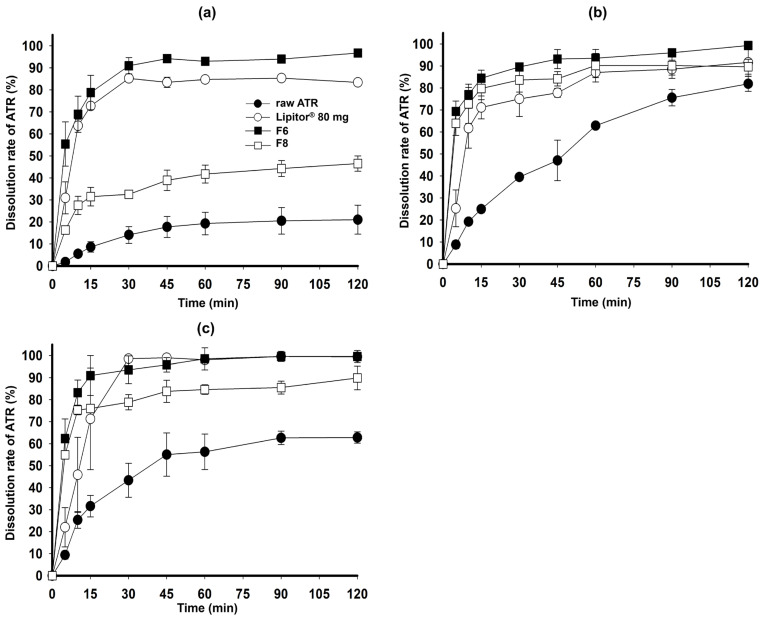
Dissolution profiles of raw ATR, Lipitor^®^ 80 mg, F6, and F10 in pH 4.0 buffer (**a**), pH 6.8 buffer (**b**), and water (**c**).

Figure 10 shows the dissolution rate of ATR in the pH 4.0 and pH 6.8 media, and water. ATR remained chemically stable in these dissolution media, and F6 exhibited a high ATR dissolution rate (>90%) in all media within 45 min. The commercial product also demonstrated a high dissolution rate at pH 1.2 and pH 6.8 but had a lower dissolution rate at the steady state compared to F6. F8, which has a high loading % of ATR (20%), showed an incomplete dissolution rate due to ATR precipitation. The optimized F6, an SGF, achieved a significantly higher dissolution improvement compared to the commercial product and raw ATR at the maximum dose due to the complementary action of PEG 200 and TPGS maximizing precipitation inhibition and solubilization. PEG 200 effectively aided in disintegration and dispersion during the dissolution test by solubilizing ATR and modulating the properties of TPGS to form a gel. However, PEG 200 had limitations in preventing precipitation of ATR and increasing the dissolution rate. On the other hand, TPGS had limitations in solubilizing ATR and demonstrating immediate release characteristics, but it was effective in inhibiting ATR precipitation and enhancing the dissolution rate during the dissolution test [29].

F6 was confirmed to be the optimized SGF through dissolution test. TPGS as a gelling agent contributed to the overall gel strength and solidification, while ATR, effectively solubilized by PEG 200, existed in the matrix as a completely homogeneous molecular dispersion. This SGF would be believed to possess a thermodynamically metastable state based on the weak crystalline structure of TPGS. Upon exposing to the aqueous medium, the SGF with low gel strength (<0.03 N), showed easy dispersion facilitated by the hydrophilic PEG 200 and TPGS, resulting in enhanced ATR dissolution. At this time, TPGS formed a microenvironmentally higher concentration than its critical micelle concentration (200 µg/mL), solubilizing atorvastatin in micellar form, thereby preventing precipitation and enhancing the dissolution rate [34]. However, if the weight ratio of ATR and TPGS were either high (F8) or low (F2), respectively, the pressure to ATR precipitation significantly increased, resulting in a decreased dissolution rate. Therefore, F6 represented an optimized in the weight ratios of ATR, PEG 200 and TPGS, and their functions in the formulation.

Additionally, the drug release data until 30 min obtained from Figure 9b and Figure 10a–c) were fitted to first-order kinetic to calculate drug release rate constants (*K*_1_). As shown in Table 2, ATR followed first-order kinetic and all coefficient of determination (*r*^2^) for all the formulations were in the range of 0.792–0.968, which means the fitting showed reliable correlation within the kinetic model. F6 showed the highest values of *K*_1_ with 1.291 at pH 1.2 where was critical dissolution medium due to ATR’s poor solubility, compared to that of the other formulations (0.608–1.105). The drug release rate constants at pH 4.0, pH 6.8 and water were in the order of F6 ≈ Lipitor^®^ 80 mg > F8 > raw ATR, which was consistent with the dissolution results.

### 2.8. X-Ray Diffraction (XRD) for the Physical Stability Analysis

**Figure 11:**
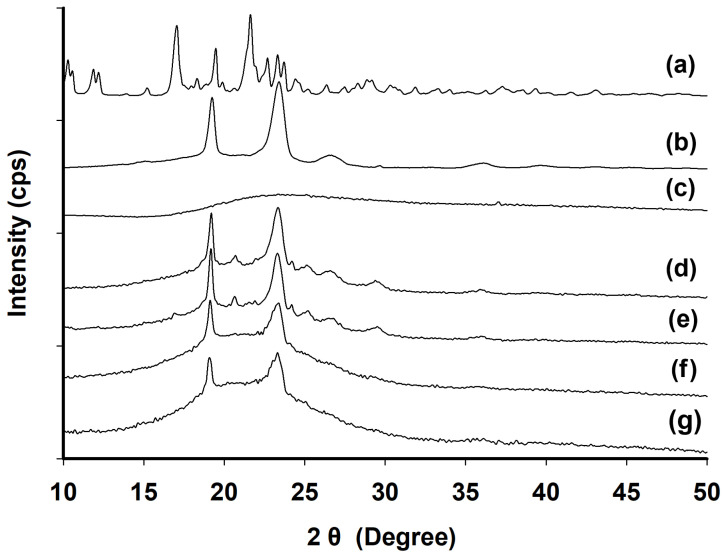
XRD patterns of raw ATR (**a**), TPGS (**b**), PEG 200 (**c**), and physical mixtures of F6 (**d**) and F8 (**e**) and F6 (**f**) and F8 (**g**) after 100 days of storage at room temperature.

XRD patterns were analyzed for raw ATR, TPGS, PEG 200, the physical mixture, F6, and F8 (Figure 11). PEG 200 showed no crystalline peaks (Figure 11c). TPGS displayed its characteristic crystalline peaks at 2θ angles of 19.24° and 23.39° (Figure 11b), which were also observed in both F6 (Figure 11f) and F8 (Figure 11g) after 100 days of storage at room temperature. In contrast, the intrinsic crystalline peaks of ATR (Figure 11a) were not detected in F6 nor F8. In the physical mixtures (Figure 11d,e), the intrinsic peak of ATR appeared between the two characteristic peaks of TPGS, indicating that ATR was partially present in a crystalline state. In summary, ATR was completely dissolved in the SGF (F6 and F8) without recrystallization over an extended period. Thus, the optimized F6 formulation from the studies remained in a physically stable amorphous form without recrystallization during long-term storage at room temperature.

### 2.9. Transmission Electron Microscopy (TEM) Analysis

**Figure 12:**
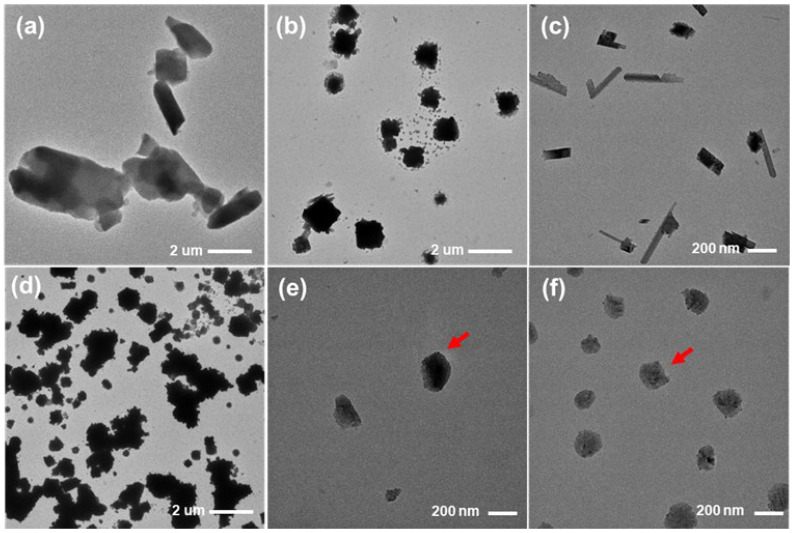
TEM images of raw ATR (**a**) treated raw ATR in pH 1.2 buffer solution (**b**), and precipitation particles obtained from dissolution test of F1 (**c**), F2 (**d**), F6 (**e**) and F8 (**f**) in pH 1.2 buffer solution.

In the dissolution test at pH 1.2, the precipitation particles collected from raw ATR and formulations were imaged using TEM and are shown in Figure 12. Raw ATR particles are rod-shaped with lengths greater than several micrometers. When immersed in the pH 1.2 dissolution medium, the collected raw ATR particles showed a reduced size due to partial sampling and the solubilization of ATR, but their overall morphology was not significantly changed (Figure 12a,b). F1 exhibited an elongated rectangular shape due to the suppression of precipitation by TPGS, with lengths ranging from 200 to 300 nm (Figure 12c). Although TPGS prevented precipitation, suggesting crystallization potential from F1, the particle size was less than 1 μm. For F2, which was solubilized with only PEG 200, many small particles 1–2 μm in size were observed, and significant precipitation occurred due to the absence of TPGS (Figure 12d). F6 and F8 showed small spherical particles less than 200 nm in size (red arrows), with F8 displaying greater number and quantity due to its higher ATR loading % (Figure 12d). Thus, F6 exhibited minimal precipitation, and its morphology was different from that of crystalline shapes. Therefore, the optimized F6 was confirmed to suppress ATR precipitation, resulting in higher dissolution rates [35].

## 3. Conclusions

ATR has very high inter-patient variation in bioavailability due to its low solubility and dissolution. Therefore, a supersaturated gel formulation (SGF) known as organogel was developed to enhance the solubility, dissolution, and physical stability of ATR. SGFs were prepared and evaluated by measuring the solubility of ATR and its dispersion solubility at pH 1.2 and pH 4.0 using PEG 200 and TPGS as a solubilizer and a precipitation inhibitor, respectively. The optimized SGF exhibited a high drug content (≥10%) and consistent and complete dissolution rates (>95%) at all pH values in the dissolution tests, including ATR and degradation products. An XRD analysis also confirmed that the SGF was physically stable without recrystallization. In conclusion, SGF loaded with ATR is a promising formulation candidate with enhanced solubility and physical stability and complete dissolution regardless of pH.

## 4. Materials and Methods

### 4.1. Materials

ATR was kindly provided by Samoh Pharm. Co., Ltd. (Seoul, Republic of Korea). The commercial ATR product, Lipitor^®^ (80 mg) was purchased from Viatris Korea Inc. (Seoul, Republic of Korea). TPGS was purchased from ISOCHEM (Vert-le-Petit, France). PEG 300, PEG 400, PEG 600, Kolliphor PS80, Solutol HS15, Kolliphor RH40 and Kolliphor EL were provided by BASF (Ludwigshafen, Germany). PEG 200 was purchased from Thermo Fisher Scientific Inc. (Waltham, MA, USA). All other chemicals were of reagent grade and used without further purification.

### 4.2. HPLC Conditions

The HPLC analysis of ATR in samples was conducted using a Waters 2695 HPLC system (Waters, Milford, MA, USA) equipped with a UV-Vis detector (Waters 2487, Waters, Milford, MA, USA). The ATR as separated using a reverse phase column (C18 column, 5 μm, 4.6 × 150 mm) (Osaka Soda, Osaka, Japan). The isocratic mobile phase consisted of acetonitrile and 0.1 M of ammonium acetate (50:50, *v*/*v*) adjusted to pH 4.0 with acetic acid. An HPLC analysis was performed with a flow rate of 1.0 mL/min. The injected volume of the sample was 10 μL, and UV detection was monitored at 270 nm. For the quantification of ATR, we established a calibration curve (1 μg/mL to 100 μg/mL, *r*² > 0.99) each time.

### 4.3. pH Solubility Test of ATR at 37 °C

The pH solubility of ATR was evaluated in pH 1.2, pH 4.0, pH 6.8, and distilled water. All tests were performed at 37 °C. Excess ATR (approximately 10 mg) was added to 10 mL of each pH buffer solution with water in a vial and shaken in a water bath at 37 °C for 24 h. Then, 1 mL of each mixture was aliquoted into a tube and centrifuged (LZ-1730R, LABOGENE, Seoul, Republic of Korea) at 15,000 rpm for 30 min. The supernatant (0.5 mL) was taken, diluted 2-fold with methanol, and then filtered through a membrane filter (0.45 μm pore size, DISMIC^®^-13HP; ADVANTEC, Tokyo, Japan). The amount of ATR in the filtrate was determined using the aforementioned HPLC conditions.

### 4.4. Solubility Test of ATR in PEGs

To test the solubility of ATR in PEG 200, 300, 400, and 600, excessive ATR (1000 mg) was added to a vial containing 1000 mg (for room temperature and 50 °C) or 600 mg (for 70 °C) of PEG. Then, the mixture was stirred at room temperature and in an oil bath at 50 °C or 70 °C for 2 h to saturate it, and each mixture was centrifuged at 15,000 rpm for 30 min (LZ-1730R, LABOGENE). The precisely weighed supernatant (500 mg) was taken into a 10 mL volumetric flask, diluted with methanol, and filtered through a membrane filter (0.45 μm pore size, DISMIC^®^-13HP; ADVANTEC). The amount of ATR in the filtrate was determined using the aforementioned HPLC conditions.

### 4.5. Dispersion Solubility Test of ATR Solution in PEGs

To prepare 10% (*w*/*w*) ATR solution in PEG, 200 mg of ATR powder was added to 1800 mg of each PEG (PEG 200, 300, 400, and 600) in a vial. Then, the mixture was stirred in an oil bath at 70 °C for 5 h, and clear 10% ATR solution in PEG was obtained. An amount of 200 mg of this solution (equivalent to 20 mg of ATR) was added to 10 mL of pH 1.2 buffer in a vial, dispersed with a vortex mixer for 1 min, and then centrifuged at 15,000 rpm for 30 min (LZ-1730R, LABOGENE). The supernatant (0.5 mL) was taken, diluted 2-fold with methanol, and filtered through a membrane filter (0.45 μm pore size, DISMIC^®^-13HP; ADVANTEC). The amount of ATR in the filtrate was determined using the aforementioned HPLC conditions.

### 4.6. Solubility Test of ATR in Surfactants

To test the solubility of ATR in surfactants, excessive ATR (100 mg) was added to a vial containing 900 mg of each surfactant and stirred in an oil bath at 70 °C for 2 h. Each mixture was centrifuged at 15,000 rpm for 30 min (LZ-1730R, LABOGENE). The precisely weighed supernatant (300 mg) was placed into a 10 mL volumetric flask, diluted with methanol, and filtered through a membrane filter (0.45 μm pore size, DISMIC^®^-13HP; ADVANTEC). The amount of ATR in the filtrate was determined using the aforementioned HPLC conditions.

### 4.7. Preparation of ATR Formulations

The total scale was 10 g for every lot of preparation, and each ingredient was weighed according to the composition%, as shown in Table 3. The precisely weighed PEG 200 and TPGS were placed into a 50 mL beaker in an oil bath at 70 °C and stirred for 1 h. Then, ATR was added to the mixture and stirred for 2 h at 70 °C to completely solubilize it, and then the liquid solution was left to cool at room temperature. There was no loss of ATR and any other ingredients, and ATR was thermally stable, allowing weight ratio % of ATR in Table 3 to be defined as the ATR loading %.

### 4.8. Solubilized Ratio of ATR in Dispersion Solubility Test of ATR Formulations

The prepared ATR formulations (accurate to 100 mg) were added to 10 mL of pH 1.2 buffer solution in a vial and dispersed with a vortex mixer for 1 min. Thereafter, 1 mL of the sample was aliquoted into tubes and centrifuged at 15,000 rpm for 30 min (LZ-1730R, LABOGENE). The supernatant (0.5 mL) was diluted 2-fold with methanol and filtered through a membrane filter (0.45-μm pore size, DISMIC^®^-13HP; ADVANTEC). The amount of ATR in the filtrate was determined using the aforementioned HPLC conditions, and the solubilized ratio (%) of ATR for each formulation (F1–F10) was calculated using Equation (1) [36].

(1) $$Solubilized\;ratio\;(\%)\;of\;ATR=\frac{The\;measured\;dispersion\;solubility\;of\;ATR\;(mg/mL)\;\times \;5\;mL\;\times \;100}{The\;calculated\;total\;amount\;of\;ATR\;(mg)\;in\;ATR\;formulation}$$

The total amount of ATR in the ATR formulation was 5.0 mg for F1, 10.0 mg for F2–F6, 20.0 mg for F7 and F8, and 30.0 mg for F9 and F10 according to the composition% in Table 3.

### 4.9. Physical Properties of ATR Formulations

The ATR formulation samples were first visually observed, and their images were obtained to determine the physical state. Secondly, thermograms of the ATR formulations were examined using DSC (DSC Q20 TA Instruments, New Castle, DE, USA). This instrument was calibrated using an indium, and the samples were scanned under nitrogen gas purging (20 mL/min). For measurements, 3–5 mg of samples was placed in an aluminum pan and covered with an aluminum lid. The heating rate was set at 10 °C/min. The results of the analysis were assessed in the range of 10~200 °C. The peak melting temperature for each endothermic curve in the thermogram was determined automatically using the DSC solution software ver. 4.5A (TA Universal Analysis 2000) [37]. Thirdly, the ATR formulations were assessed for their gel strength. The ATR formulations were uniformly distributed in the wells of 96-well plates and analyzed using a texture analyzer (TAXTplus; Stable Micro Systems, Godalming, UK) equipped with a spherical puncturing probe (diameter 5 mm). The pre-test speed was set at 0.1 mm/s with a trigger force of 0.005 N and a test speed of 0.2 mm/s. Gel strength was defined as the peak positive force (N) during the passage of the plunger through the gel [38].

### 4.10. Dissolution Studies

The dissolution test was performed using a dissolution tester (708-DS Agilent Technologies Inc., Santa Clara, CA, USA) at 50 rpm and 37 ± 0.5 °C using 900 mL of pH 1.2, pH 4.0, and pH 6.8 buffer solutions and water. The test was performed according to the paddle method using the dissolution medium. Dissolution samples except for raw ATR were heated to 70 °C to liquefy them and lower the viscosity and were manually filled into appropriately sized gelatin capsule shells to the predetermined amount; F1 (1736.0 mg), F2 (868.0 mg), F6 (868.0 mg), F8 (434.0 mg), and F10 (289.3 mg). These samples were cooled down at room temperature for 24 h. Raw ATR was manually filled into #1 gelatin capsule shell with 86.8 mg of ATR. The prepared samples with the amount of formulation equivalent to 86.8 mg of ATR (=80.0 mg as atorvastatin free form) were placed in a dissolution tester. [14,15]. The samples were aliquoted at 5 mL using a syringe at predetermined time intervals (5, 10, 15, 30, 45, 60, 90, and 120 min). The aliquoted sample was diluted 2-fold with methanol and filtered through a membrane filter (0.45 μm pore size, DISMIC^®^-13HP; ADVANTEC). The amount of ATR in the filtrate was determined using the aforementioned HPLC conditions. At pH 1.2, ATR was unstable and produced major degradation materials, including Imp-A1, Imp-H, and Imp-J, in the HPLC chromatogram, and the dissolution rate of the sum of the ATR and degradation materials was also measured using the previously reported relative response factor of the degradation materials over ATR [39]. The response factors for these three degradants were known to be 1.00 in common and the simple total sum of peak areas for ATR, Imp-H, Imp-J, and Imp-A1, in the HPLC chromatogram was used for the measurement of dissolution rate of ATR and degradants (%) at pH 1.2.

### 4.11. XRD Pattern Analysis

XRD patterns were measured using an X-ray diffractometer (Rigaku, Ultima IV, Tokyo, Japan) equipped with a Linxeye 1-D detector. Each sample was added to the grid, and their diffraction patterns were measured using a Cu/K radiation source (40 kV and 40 mA) with the acquisition time of 0.2 s per step. The scanning range was 10~50 in the 2θ range.

### 4.12. TEM Analysis

The particle shape and size were analyzed using a transmission electron microscope (Talos L120C, Thermo Fisher Scientific Inc., Waltham, MA, USA). The sample was prepared by taking a sample 2 h after the start of the dissolution test, adding it dropwise to a carbon-coated TEM grid (TF300C, TMALAB, Goyang, Republic of Korea), and drying it for 24 h. All measurements were performed at a voltage of 120 kV at room temperature.

### 4.13. Drug Release Kinetics

The drug release data was fitted to kinetic models of first-order, and the coefficient of determination (*r*^2^) was indicative of best-fit model to describe drug release kinetics [38].

## Figures and Tables

**Table 1:** pH solubility of ATR at 37 °C.

Test Solution	Solubility (μg/mL)
pH 1.2	6.63 ± 0.24
pH 4.0	12.81 ± 1.16
pH 6.8	158.35 ± 4.58
water	66.30 ± 0.89

Data are expressed as the mean ± standard deviation (*n* = 3).

**Table 2:** Drug release rate constant (first-order rate constant, *K*_1_) and coefficient of determination (*r*^2^) of ATR for representative formulations.

Formulation	pH 1.2		pH 4.0		pH 6.8		Water	
	*K*_1_	*r* ^2^	*K*_1_	*r* ^2^	*K*_1_	*r* ^2^	*K*_1_	*r* ^2^
Raw ATR	0.608	0.833	0.752	0.892	1.040	0.982	1.092	0.968
Lipitor^®^ 80 mg	0.988	0.864	1.291	0.895	1.273	0.899	1.325	0.955
F6	1.291	0.917	1.283	0.829	1.278	0.792	1.305	0.812
F8	1.105	0.952	1.004	0.879	1.260	0.794	1.253	0.805

**Table 3:** Composition ratio of ATR formulations.

Formulation	Composition Ratio (%, *w*/*w*)		
	ATR	PEG 200	TPGS
F1	5	0	95
F2	10	90	0
F3	10	85	5
F4	10	80	10
F5	10	70	20
F6	10	60	30
F7	20	60	20
F8	20	55	25
F9	30	55	15
F10	30	45	25

## Data Availability

Data are contained within the article.
